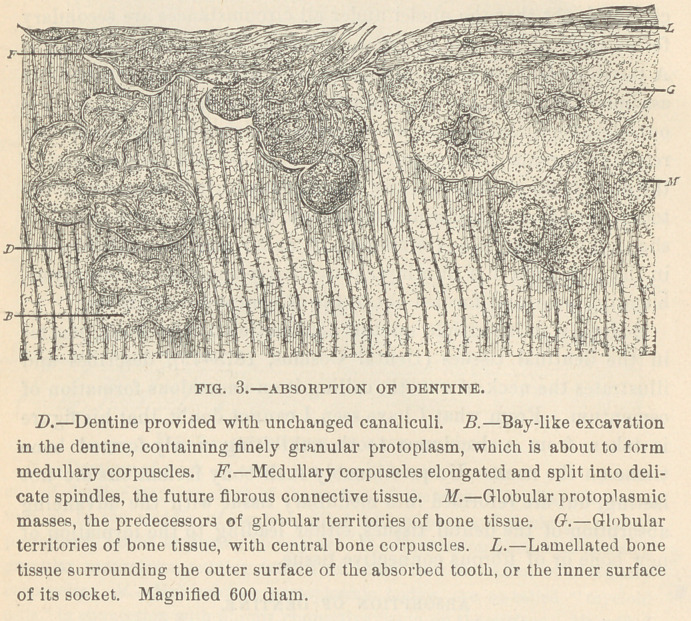# Microscopical Studies upon the Absorption of the Roots of Temporary Teeth

**Published:** 1884-07

**Authors:** Frank Abbott


### MICROSCOPICAL STUDIES UPON THE ABSORPTION OF THE ROOTS OF TEMPORARY TEETH.

BY FRANK ABBOTT, M. D.

Whitney Memorial Prize Essay. Read Before the Dental Society of the State of New York, at its Sixteenth Annual Meeting, May 14 and 15, 1884.

The fact that temporary teeth, previous to the appearance of the permanent ones, are considerably reduced in size, often lacking roots, often consisting only of a thin shell, and exhibiting a corroded festooned surface, has long since attracted the attention of observers. It has never been doubted that a persistent, though graded, irritation causes the absorption; but what the real cause of this irritation is we cannot tell. The idea that in all cases the pressure of the growing permanent tooth is the direct cause must be abandoned, since clinical observation shows that the absorption of a temporary tooth may take place though far distant from the permanent one; nevertheless, we maintain that the growth of the latter, indirectly at least, causes the irritation, and consequent absorption.

The assertion of Tomes that it is due to the presence of freely vascularized papilla does not explain the decrease of the dental tissues, for the papilla is nothing but medullary tissue, such as we meet with in any part of the organism where one tissue is about to change into another. Such a papilla can be the cause of the absorption, as well as its result. Another assertion, that the medullary cells eat out the dental tissues by their active growth, or by their ameboid motions, is insufficient for the explanation of the loss of the lime-salts in the dental tissue, the presence of circular or semi-circular excavations and bays, so characteristic of the melting process of the cementum and dentine of deciduous teeth.

Since we know that pieces of dead bone or ivory may be absorbed with figures similar to those found on the surface of temporary teeth, the idea possibly becomes admissible that, owing to the presence of an acid, first the lime-salts are dissolved out within certain territories of the dead bone tissue in a merely chemical or passive way, whereupon the soft medullary tissue penetrates the spaces thus established. Quite different, however, will be the conception of this process if we bear in mind that the temporary teeth, as well as the permanent ones, are made up of living tissues, and an active participation of these tissues must be expected in the process of transformation of the dental into that of medullary tissue. As the process of absorption is closely allied to the process of inflammation, and active changes of the dental tissues have been proven to follow inflammation beyond any doubt, we may *a priori* expect such changes of the bone tissues of the temporary teeth in the process of absorption also. I shall try to prove in this paper that such changes really do occur. In the light of the most advanced modern views concerning the structure of the dental tissues, we consider cementum, dentine, and enamel as endowed with properties of life, or, in other words, as pervaded by living matter in the shape of an extremely delicate reticulum. In this view not only the cement corpuscles and their coarser offshoots contain living matter in the shape of so-called "granular protoplasm," but the whole basis-substance present between the cement corpuscles is alive also, only the minute meshes of the living reticulum holding a gluey basis-substance saturated with lime-salts. In the dentine, not only the tenants of the dentinal canaliculi (the dentinal fibers) are alive, but the whole mass of gluey and calcified basis-substance between the dentinal canaliculi is also living. The same holds good for the enamel, in which the delicate fibrilæ between the enamel prisms have positively been proven to be living matter, but the prisms are pierced by living matter, though the latter has not been demonstrated directly, but indirectly in morbid changes of the enamel. Of the cementum, we know that each cement corpuscle occupies the center of a more or less globular territory of basis-substance. If, therefore, circular fields of absorption appear in the process of inflammation and absorption of the cementum, we can readily trace these territories in following out the portion affected by the process of absorption. But how shall we explain the bay-like excavations in the dentine and enamel so often seen in reduced temporary teeth, where there is nothing known of territories? Here the first difficulty sets in, due to the lack of knowledge of the history of the development of dentine and enamel. Czermak's interglobular spaces indicate the presence of such territories in the dentine, the presence of which, however, can be proven only after accurate researches in the history of development.

Granted that the dissolution of lime-salts takes place in globular territories in the dental tissues, the next question will be, how do the medullary elements appear in such spaces? Do they migrate or penetrate from without, or do they originate, in part at least, from the living material present in all dental tissue?

### ABSORPTION OF CEMENTUM.

The process of absorption of a provisional tooth begins on the cementum of the roots. The latter exhibits before the beginning of this process the features of cementum of permanent teeth. Primarily, the absorption is marked by the appearance of the well-known fields so commonly met with in the process of osteitis, that is, excavations on the surface, either semi-circular or composed of a varying number of semi-circular festoons, all of which are filled with medullary elements, multinuclear bodies, or a delicate myxometa, in part a bony, in part fibrous connective tissue, blending with the adjacent myxometous or fibrous pericementum. The communication of the excavations with the pericementum is either widely gaping or through a narrowed neck on the surface of the cementum. Sometimes, however, in the sections the excavations appear isolated, without any communication with the surface, which latter instance, however, will certainly not entitle us to deny the existence of such a communication on a plane above or below that of the section. In the excavations the cementum is unquestionably reduced first into medullary, afterward into myxometous or fibrous tissue. By closely watching excavations of a more recent date at the periphery of those in communication with the pericementum, we notice that the lime-salts and the basis-substance proper are missing, and are replaced by a uniformly granular protoplasm, or a varying number of faintly marked medullary elements, each of which may contain a central nucleus. We can trace a gradual change of the tissue of cementum from a dissolution of lime-salts to the appearance of a mass of granular protoplasm, and at last to the formation of medullary corpuscles. The circular shape of the excavation is in all cases undoubtedly due to a dissolution of the lime-salts, and afterward of the basis-substance proper, within the territory of a cement corpuscle. Sometimes we see an enlargement of the lacuna and the cement corpuscle itself, the latter splitting up into a varying number of glistening lumps, which are readily stained by an ammoniacal solution of carmine. In other instances the whole territory of a cement corpuscle is transformed into protoplasm, and the reappearance of such protoplasm is traceable through broad offshoots to neighboring cement corpuscles. In a third instance a varying amount of the territory has assumed a delicate fibrous appearance, caused by an early grouping of the medullary corpuscles into fibrillæ. In neither of these instances will it be doubted that the cement corpuscles themselves, or the living matter held in their territories, have in an active way taken part in the reappearance, first of protoplasm, and afterward of medullary corpuscles. The theory that immigrated medullary corpuscles, or "leucocytes," have replaced the former cement tissue must be abandoned as soon as we can trace a gradual transformation of the tissue of the cementum into medullary tissue. The latter immediately assumes the characteristic features of a myxometous or fibrous connective tissue, in connection with the pericementum (Fig. 1).

From this point of view there is no difficulty in explaining the appearance of multinuclear bodies, so-called "myelo-plaxes," in the dissolved territories. We know that such formations represent a stage of development of cementum, and they simply reappear as soon as the basis-substance of an already-formed cementum is dissolved or liquefied. In fact, nothing else is required but a reformation of basis-substance and its recalcification, in order to reproduce new bony tissue, such as we often meet with on the periphery of absorbed cementum.

The result of the absorption is, next a myxometous or fibrous connective tissue freely supplied with newly-formed blood vessels.

In this tissue an active new formation of bony trabeculæ of bone takes place, characterized by the presence of large and irregular bone corpuscles. The widened socket, or dissolving surface of an absorbing provisional tooth, is not infrequently filled with newly formed bone. The newly formed layer of cementum in part shows circular fields (territories) of bone tissue, each of which may contain a varying number of bone corpuscles, or there is a uniform reduction of the original cementum, the boundary of which is made up by regularly arranged medullary corpuscles, so-called "osteoblasts."

On the neck of the tooth the excavations penetrate not only the layer of the cementum proper, but also the layer of the subjacent dentine, which we know to be destitute of dentinal canaliculi. Here again we observe at first a dissolution of the basis-substance in globular fields, which appear filled at first with a finely granular protoplasm, lacking nuclei, afterward with usually nucleated medullary corpuscles, and at length with a slightly fibrillated tissue, the latter undoubtedly originating from a splitting of the medullary corpuscles into a number of delicate spindles. The surface of the neck of the tooth likewise exhibits the characteristic bay-like excavations which are filled with nucleated medullary corpuscles, or with multinuclear protoplasmic bodies. From what I have seen, I cannot doubt that the nuclei under all circumstances are secondary formations, and cannot be regarded as the future bone corpuscles. A territory of bone tissue will form only after the protoplasm has assumed a uniform granulation, and the bone corpuscles will develop out of this protoplasm, by an increase of living matter at certain regular intervals. The result of the absorption and reappearance of the embryonal condition of the tissues, constituting the neck of the tooth, results in the formation of new bone tissue, either in the shape of globular fields (territories) of newly-formed bone tissue, or in the formation of a thin layer of regularly lamellated bone tissue, blending with that formed out of the cementum of the roots.

Bodecker, in his article on the distribution of the living matter in the dentinal tissues (Dental Cosmos, 1878–79), describes and illustrates the neck of a tooth, calling it an anomalous formation of cementum. From what I have seen I cannot doubt that his figure is taken from a deciduous tooth, exhibiting newly-formed bone tissue on the neck. Unquestionably such bony formations are not lasting, but are reformed into medullary tissue with the advancing absorption of the dental tissues, either leading to the formation of new bone or of fibrous connective tissue.

### ABSORPTION OF DENTINE.

The most striking features in the dentine of deciduous teeth are the bay-like excavations on the surface after a complete disappearance of the covering cementum. The excavations contain medullary corpuscles, multinuclear bodies, or fibrous connective tissue, in connection with the surrounding pericementum, or periodontium. The appearance of such fields in the dentine gave rise to the theory that a foreign tissue grows into the dentine, destroying it in the manner in which dead bone is destroyed. If, however, we bear in mind that the tissue of dentine is composed of globular territories, the same as that of bone, we at once are in the position to understand the striking appearance of globular fields of absorption in the dentine. The question can only be, is the dentine absorbed in a merely passive way, or does it share in the formation of medullary tissue, so long, at least, as it is the seat of life in itself? My researches strongly point in favor of an answer in the latter direction. In several instances I have been able to trace a slight widening of the dentinal canaliculi on the border of the fields of absorption, with an increase of living matter in the canaliculi. Such features are very common in the process of caries, where the dilatation of the canaliculi at the expense of the intervening basis-substance, and a new formation of medullary elements out of the tenants of the canaliculi, is a very common occurrence, provided that the caries attacks the living dentine. Circumscribed bay-like excavations, such as are common in the absorption of dentine, I have frequently met with in caries.

Sometimes such excavations, in caries, form independently of the surface destruction, and with the lower powers of the microscope even a well-versed eye, under these circumstances, would experience difficulty in discriminating between carious destruction and absorption of temporary dentine. Higher powers, to be, sure, reveal the presence of micro-organisms in the former process, which are lacking in the latter. The first step seems to be identical in both instances; a dissolution of the lime-salts, or a displacement of the lime-salts, by the liquefaction of the glue giving basis-substance. After this, medullary elements arise out of the liquefied dentine which are destined to decay in caries, and, on the contrary, proliferate in the process of absorption, with the result of the new formation of medullary tissue. In caries the process of softening, or the removal of the calcified basis-substance, results in the death and putrefaction of the tissue; that is, its replacement by micro-organisms. In the process of absorption, on the contrary, it ends in an active proliferation of medullary tissue, with the non-interference of micro-organisms.

The result of the latter process is the same whether it occurs on the cementum, on the neck of the tooth, or in canalicularized dentine. The newly-formed medullary tissue consists either of single medullary corpuscles, usually with one oblong and faintly-marked nucleus, or in the shape of larger protoplasmic masses with a varying number of oblong nuclei, so called "myeloid cells." At the border of the bays I have sometimes been able to trace delicate thorny projections from the multinuclear masses into the unchanged basis-substance of the dentine. Sometimes I have seen broad offshoots of the multinuclear bodies penetrating the widened dentinal canaliculi, and in direct union with the dentinal fibers. The latter feature, especially, seems to point strongly toward an organic connection between the unchanged and the dissolved out dentine.

The multinuclear bodies are, as is well known, the future territories of bone tissue, and therefore predecessors of bone tissue. The formation of bony territories can easily be traced on the surface of absorbed dentine. Just as in normal development of bone tissue globular territories first appear, and afterward lamellated bone tissue, so also in absorbed dentine, first multinuclear bodies, afterward globular territories of bone tissue, and at last lamellated bone tissue forms on the surface, the latter producing, in many instances, a continuous layer of bone all around the absorbed tooth. This feature is correctly observed and described by Tomes.

As to absorption of the enamel I can say but little. It is well known that bay-like excavations are seen in this tissue, in no way differing from those of cementum and dentine. From this fact it would follow that all the theories hitherto advanced with regard to the development of enamel must be erroneous, and there must be an arrangement in the enamel forming tissue leading to the production of territories in a manner similar to that of dentine and cementum. It seems that the process of destruction, starting on the surface of the enamel, is in all instances caries, and not absorption. The latter process attacks enamel only from within. After most of the dentine has been absorbed and transformed into myxometous tissue, the enamel is attacked and thinned to a varying degree by the same process. Thus it becomes intelligible that the shell of enamel left, is coated by a layer of lamellated dentine, which is continuous all around the remnants of the provisional tooth.

Since we know that enamel is a live tissue in a living tooth, we may anticipate its reduction into medullary tissue in the process of absorption. Whether or not such a breaking down of enamel occurs and its consequent participation in the formation of bone tissue, I am unable to say.

## Figures and Tables

**Fig. 1. f1:**
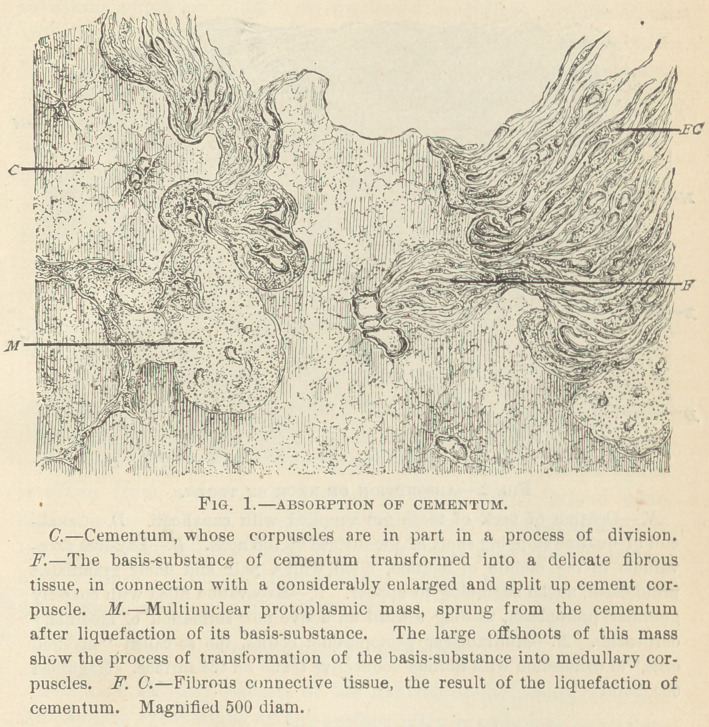


**Fig. 2. f2:**
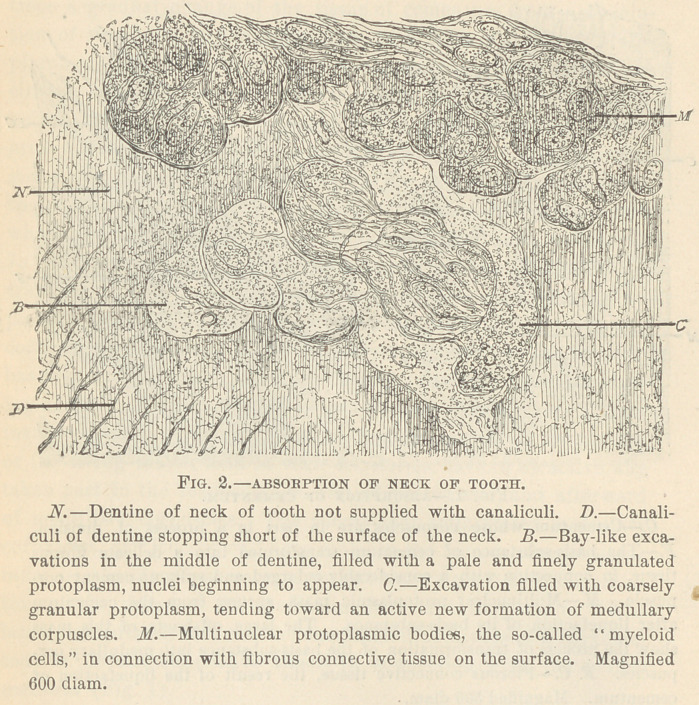


**Fig. 3. f3:**